# EMAIL-HF: Can Digital Strategies Shorten the Path from Scientific Evidence to Clinical Practice in Heart Failure?

**DOI:** 10.1007/s10741-026-10691-7

**Published:** 2026-11-07

**Authors:** Monica T. A. Albuquerque, Patricia O. Guimaraes, Caio A. M. Tavares, Vagner Madrini, Eduardo B. Martins, Kalliopi Keramida, Dike Ojji, Kaveh Hosseini, Luca Monzo, Francesca Musella, Yasuhiko Sakata, Mario Enrico Canonico, Tamas Alexy, Jan Biegus, Savina Nodari, Andrew Ambrosy, Mitja Lainscak

**Affiliations:** 1 Hospital Israelita Albert Einstein, São Paulo, Brazil; 2 Cardiology Department, Oncological Hospital, General Anti-Cancer, Agios Savvas, Athens, Greece; 3 University of Abuja Teaching Hospital, Gwagwalada, Abuja, Nigeria; 4 Copenhagen University Hospital – Herlev and Gentofte, Copenhagen, Denmark; 5 Cardiovascular Non-Invasive Imaging Research Laboratory (CIRL), Hellerup, Denmark; 6 CHRU de Nancy, Centre d’Investigation Clinique Plurithématique 1433, INSERM, Nancy, France; 7 Université de Lorraine, INSERM U1116 – DCAC, Nancy, France; 8 U.O.C. Cardiology, P.O. Santa Maria delle Grazie, Pozzuoli, NA Italy; 9 Division of Cardiology, Department of Medicine, Karolinska Institutet, Stockholm, Sweden; 10 Department of Clinical Medicine and Development, National Cerebral and Cardiovascular Center, Suita, Japan; 11 CPC Clinical Research, Aurora, CO USA; 12 Department of Medicine, Univerity of Colorado, Aurora, CO USA; 13 University of Minnesota, Minneapolis, MN USA; 14 Department of Cardiology, Clinical Department of Intensive Cardiac Care, Faculty of Medicine, Institute of Heart Diseases, Wroclaw Medical University, Borowska 213, Wroclaw, 50-556 Poland; 15 Department of Medical and Surgical Specialities, Univerity of Brescia, Brescia, Italy; 16 Department of Cardiology, Kaiser Permanente San Francisco Medical Center, San Francisco, CA USA; 17 Division of Research, Kaiser Permanente Northern California, Pleasanton, CA USA; 18 Faculty of Medicine, University of Ljubljana, Ljubljana, Slovenia; 19 General Hospital Murska Sobota, Murska Sobota, Slovenia

**Keywords:** Heart failure, SGLT2 inhibitors, Digital strategy

## Abstract

A substantial gap persists between scientific evidence and clinical practice in heart failure (HF). Digital solutions have emerged as promising strategies to accelerate the implementation of evidence-based therapies. This mini-review examines the results of the EMAIL-HF study, a registry-based, randomized, controlled, parallel-group clinical trial conducted in Denmark that included patients aged 20 years or older with a diagnosis of HF irrespective of left ventricular ejection fraction, who were not receiving SGLT2 inhibitors (SGLT2i). Participants were randomized to usual care or a digital strategy consisting of an e-mailed letter with information on SGLT2i, followed by contact with an HF specialist. The primary endpoint was the proportion of patients redeeming a prescription for dapagliflozin or empagliflozin within 6 months of randomization and the secondary endpoint was a composite of time to first HF hospitalization or death from any cause. The digital strategy significantly increased SGLT2i initiation within 6 months, although no difference in HF hospitalization or all-cause mortality was observed during the 24-month follow-up. Whether these strategies can translate modest improvements in treatment uptake into sustained, scalable, and cost-effective gains across diverse healthcare systems remains uncertain.

A substantial gap persists between the generation of new scientific data and the adoption of evidence-based treatment recommendations into routine clinical practice [1], even in high-mortality conditions such as heart failure (HF) [2, 3]. For instance, despite the robust data supporting the use of sodium–glucose cotransporter 2 inhibitors (SGLT2i) (Fig. 1) and a very large eligibility in real world population [4, 5], this therapeutic class remains underutilized in real-world settings. Multiple factors may contribute to this evidence-to-practice gap, including clinical inertia, patient and healthcare professional-related barriers, as well as socioeconomic factors, frailty, aging, and polypharmacy [2, 3]. Against this backdrop, the rapid digital transformation of society is reshaping clinical practice, and HF care is no exception.

Herein, the EMAIL-HF trial [6] explores how information from existing datasources can be crossreferenced with current guideline recommendations using conventional and modern technologies in order to identify elegible patients for SGLT2i. The highly developed digital and registry infrastructure in Denmark provides an important setting for this study.

The EMAIL-HF trial results were presented at the European Society of Cardiology Congress in Munich, in 2026. The trial findings are of particular interest to the scientific community and health policy stakeholders. Thus we discuss the main trial results and potential future directions in this report.

EMAIL-HF was a registry-based, randomized, controlled, parallel-group, clinical trial conducted in Denmark. It enrolled patients aged 20 years and older with HF diagnosis within the past 10 years, irrespective of left ventricular ejection fraction (LVEF), who were not receiving treatment with SGLT2i therapy (Fig. 2). Participants were randomized to receive digital intervention (in addition to usual care) or usual care only. Patients assigned to the intervention group received a digital letter sent by e-mail with detailed information about SGLT2i and an invitation to be evaluated by a HF specialist. In case of a positive reply, they were scheduled for a 30-min phone call with a HF specialist, who scrutinized the available patient information using the e-records and determined if they were actually eligible for SGLT2i therapy or if other tests were required before such decision could be made. Those prescribed an SGLT2i continued follow-up in either specialized outpatient clinics or primary care clinics, while those in the control group received usual care within the Danish healthcare system. The primary endpoint was the proportion of patients redeeming a prescription for dapagliflozin or empagliflozin within 6 months of randomization and the secondary endpoint was the composite of time to first HF hospitalization or death from any cause. Follow-up was performed using data from various registries with comprehensive and detailed health-related and socioeconomic information [7]. The primary analysis followed the intention-to-treat principle. Further details on the study design have been published previously [6].

EMAIL-HF identified 12,592 patients with HF, of which 3,810 (30%) were using any SGLT2i. Considering that the trial was conducted between May 2023 and June 2024 and that SGLT2i received a Class I recommendation for HFpEF only in 2023 [8], it may be difficult to interpret this number without a breakdown of SGLT2i use according to LVEF. Other reasons for trial exclusion were exempt from digital post (an official digital communication channel between individuals or companies and the government), type 1 diabetes and cancer within the past year. For the 5,996 participants included, the mean age was 71 years, 33% were women and the mean HF duration was 65 months. At baseline, approximately 70% were on a beta-blocker, 80% on angiotensin-converting enzyme inhibitor/ angiotensin II receptor blocker or sacubitril-valsartan, 56% on diuretics and 33% on one of the mineralocorticoid receptor antagonists. Approximately 40% of randomized patients had a history of HF hospitalisation, which further highlights the implementation gap of evidence-based medicine even after acute decompensations, consistent with data showing that only 20.2% of eligible patients hospitalized with HFrEF received an SGLT2i at discharge in a nationwide U.S. cohort [9].

The response rate to the invitation e-mail in the intervention group was 60%. Overall, a third of responders had SGLT2i recommended, a third had this therapy not recommended and the final third could not be reached or preferred to discuss with their general practicioner before evaluation. Eventually, 18.6% patients in digital strategy arm and 8.2% in the usual care arm received prescription for SGLT2i within 6 months of randomization (risk ratio 2.28, 95% CI: 1.98–2.63, *p* < 0.0001). No difference was observed in the composite secondary outcome of time to first HF hospitalisation or death between study groups during the 24-month follow-up (hazard ratio 0.92, 95% CI 0.81 to 1.06). Among patients who were initiated on dapagliflozin or empagliflozin during the follow-up, each prespecified serious safety event, including ketoacidosis, urosepsis, and fall resulting in fracture, was reported in fewer than five patients in each cohort.

Considering the universal recommendation of using SGLT2i for the management of HF, the modest increasing uptake over the 6 months follow-up warrant attention. Data from the Swedish Heart Failure Registry showed a rapid increase in SGLT2i use between 2022 and 2024, up from 78% to 89% in HFrEF, 54% to 80% in HFmrEF, and 41% to 80% in HFpEF [10]. This suggests particularly rapid uptake following the expansion of guideline recommendations across the EF spectrum in a population similar to that studied in the EMAIL-HF. Similarly, in a nationwide French HF remote monitoring cohort, SGLT2i prescription at program entry increased markedly between 2021 and 2024 following pivotal trials and guideline updates, although use remained lower in patients with chronic kidney disease [11]. Taken together, these findings suggest that the incremental value of digital strategies may be limited in these settings, since increases in prescription were seen without targeted interventions. Further analyses of the reasons underlying non-prescription in the EMAIL-HF trial will be important and should be reported in future publications, particularly given that documented contra-indications to SGLT2i may account for as few as 3.7% of patients not receiving this therapy [12]. Given that some patients preferred to discuss pharmacotherapy with their general practitioner, it might be worth exploring whether a more integrated implementation pathway, adding, for example, protocolized active engagement of primary care providers along with targeted educational strategies, may further improve treatment uptake. Indeed, digital tools should be viewed as a complementary strategy to facilitate the adoption of disease-modifying therapies by both healthcare providers and patients, rather than as a stand-alone solution to the implementation gap.

In EMAIL-HF no between-group difference was observed in the secondary composite outcome of time to first HF hospitalisation or death. However, the trial was not powered to assess whether the digital intervention would reduce clinical events. The modest increment in SGLT2i prescription in the intervention group compared with the usual care group may have been insufficient to produce significant differences in the rates of outcome events. These considerations may partly explain the neutral secondary outcome and underscore the importance of assessing sustained treatment use when evaluating the clinical impact of digital implementation strategies. Data on treatment adherence and persistence will provide further insights when the trial results are published.

From an economic perspective, the value of digital implementation strategies depends not only on their ability to increase treatment initiation but also on whether these gains lead to treatment maintenance, clinical benefits [13–16] and less healthcare utilization. In 2022, the STRONG-HF (Safety, tolerability, and efficacy of up-titration of guideline-directed medical therapies for acute heart failure) study demonstrated that an intensive guideline-directed medical therapy (GDMT) optimization program following hospitalization for acute HF—which at the time did not include SGLT2i—significantly reduced clinical events and was potentially cost-effective across countries with diverse socioeconomic and healthcare settings [17]. Although SGLT2i have been shown to be cost-effective overall, particularly in HFrEF [18], these findings may not be generalizable to all strategies aimed at improving treatment uptake. Cost-effectiveness analyses using EMAIL-HF data could help determine the cost per patient initiated on SGLT2i therapy and the level of treatment uptake required to generate a meaningful benefit for the health-care system. In parallel, large-scale initiatives such as JACARDI highlight the importance of coordinated approaches to implementing evidence-based interventions across European healthcare systems [19], underscoring the need to evaluate whether such approaches can deliver meaningful and economically sustainable improvements in clinical practice.

Importantly, digital strategies in HF may extend beyond treatment initiation to support longitudinal disease management, including medication adherence, GDMT optimization, monitoring, and follow-up. In healthcare settings where national registries and integrated digital communication infrastructure are less established than in countries such as Denmark, digital strategies need to be tailored to local healthcare structures and resources. A recent metanalysis showed that digital health interventions (telemedicine, SMS-based systems, mobile applications) across low- and middle-income countries settings were associated with significant reductions in all-cause and HF-specific hospitalisations, along with lower all-cause mortality. Some limitations of this analysis should be noted: there was no baseline report of digital literacy and studies with small sample sizes and with unblinded outcome assessment were included [20]. Interestingly, even in highly digitalized healthcare systems, such as Denmark, digital literacy and access to digital communication remain important barriers to participation in these trials. In the EMAIL-HF trial, exemption from Digital Post was the second most common reason for exclusion, surpassed only by existing SGLT2i use. The exemption from Digital Post may reflect a range of barriers, including physical or cognitive disabilities and lack of access to digital devices or internet connectivity [21], what may select a younger and more digitally engaged population. These findings highlight that well-established digital infrastructure does not necessarily ensure universal digital access or literacy, reinforcing the need to adapt digital strategies to patients’ capabilities and local healthcare contexts.

The adoption of new evidence-based therapies into clinical practice is a complex process that inevitably takes time. The EMAIL-HF trial showed a statistically significant but modest improvement in treatment uptake, indicating that digital interventions alone may not be sufficient to substantially accelerate treatment implementation. Future research should assess the reproducibility of these findings across different healthcare systems, the sustainability of improvements in treatment uptake, whether these improvements translate into clinical benefits, and the cost-effectiveness and scalability of such strategies.

**Fig. 1:**
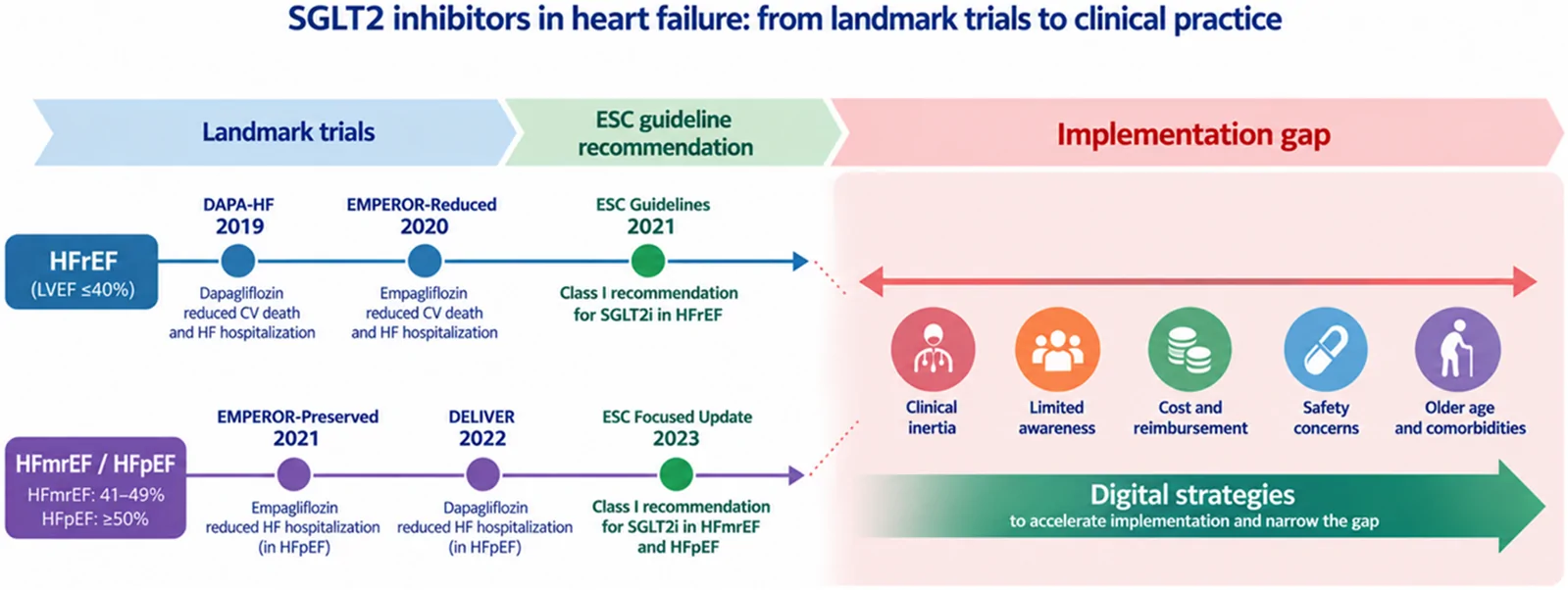
Timeline for clinical evidence on SGLT2i. HF: Heart Failure; HFrEF: Heart Failure with Reduced Ejection Fraction; HFmrEF: Heart Failure with Mildly Reduced Ejection Fraction; HFpEF: Heart Failure with Preserved Ejection Fraction; ESC: European Society of Cardiology

**Fig. 2:**
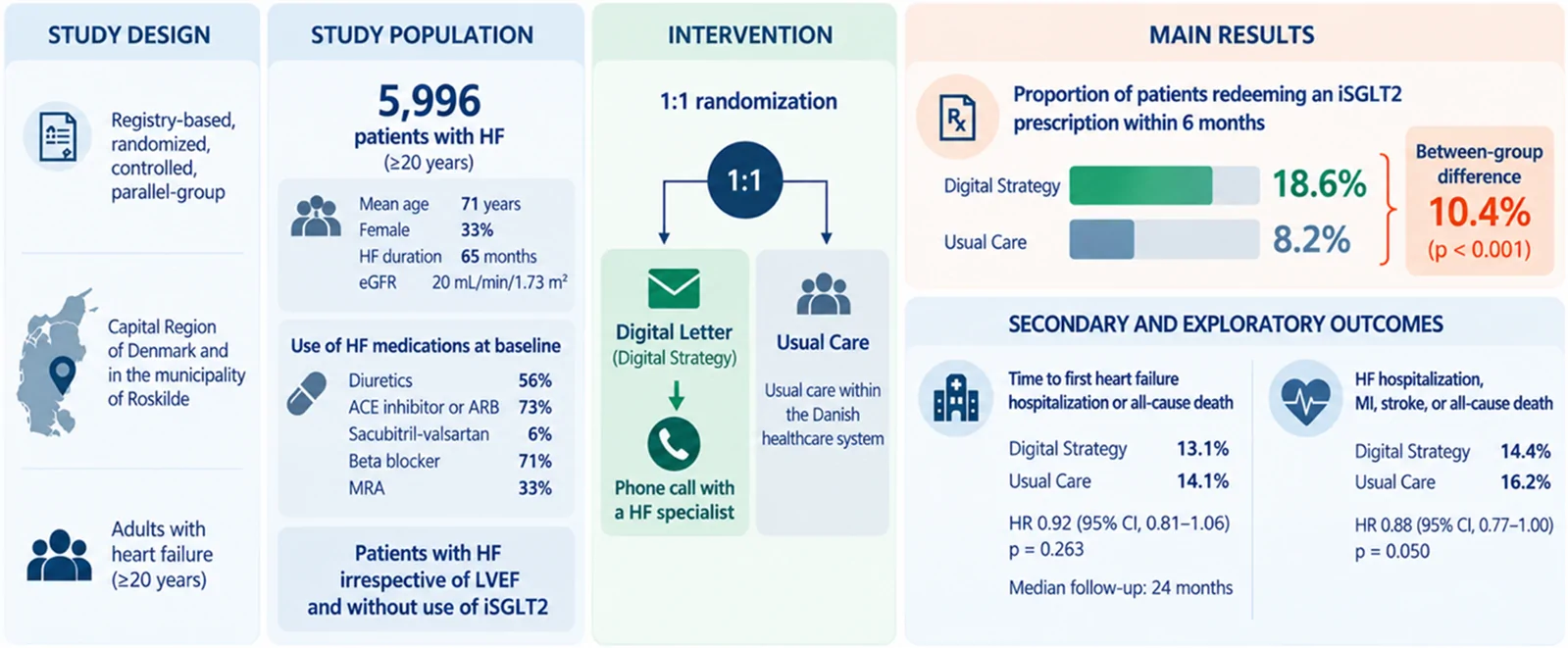
EMAIL-HF study design and main results. HF: Heart Failure; eGFR: estimated Glomerular Filtration Rate; ACE: Angiotensin-Converting Enzyme; ARB: Angiotensin II Receptor Blocker; MRA: Mineralocorticoid Receptor Antagonist;HR: Harzard Ratio

## Data Availability

No datasets were generated or analysed during the current study.

## Declarations

### Use of generative artificial intelligence

During manuscript preparation and revision, generative artificial intelligence tools were used to assist with language editing, stylistic refinement, readability and development of the figures. The authors take full responsability for the content of the publication.
